# The etiology of Ebola virus disease-like illnesses in Ebola virusnegative patients from Sierra Leone

**DOI:** 10.18632/oncotarget.8558

**Published:** 2016-04-02

**Authors:** Wen-Gang Li, Wei-Wei Chen, Lei Li, Dong Ji, Ying-Jie Ji, Chen Li, Xu-Dong Gao, Li-Fu Wang, Min Zhao, Xue-Zhang Duan, Hui-Juan Duan

**Affiliations:** ^1^ 302 Military Hospital of PLA, Beijing, China; ^2^ Sierra Leone-China Friendship Hospital, Freetown, Sierra Leone

**Keywords:** Ebola virus disease (EVD), EVD-suspected cases, malaria, HIV, Lassa fever

## Abstract

During the 2014 Ebola virus disease (EVD) outbreak, less than half of EVD-suspected cases were laboratory tested as Ebola virus (EBOV)-negative, but disease identity remained unknown. In this study we investigated the etiology of EVD-like illnesses in EBOV-negative cases. From November 13, 2014 to March 16, 2015, EVD-suspected patients were admitted to Jui Government Hospital and assessed for EBOV infection by real-time PCR. Of 278 EBOV negative patients, 223 (80.21%), 142 (51.08%), 123 (44.24%), 114 (41.01%), 59 (21.22%), 35 (12.59%), and 12 (4.32%) reported fever, headache, joint pain, fatigue, nausea/vomiting, diarrhea, hemorrhage, respectively. Furthermore, 121 (43.52%), 44 (15.83%), 36 (12.95%), 33 (11.87%), 23 (8.27%), 10 (3.60%) patients were diagnosed as infection with malaria, HIV, Lassa fever, tuberculosis, yellow fever, and pneumonia, respectively. No significant differences in clinical features and symptoms were found between non-EVD and EVD patients. To the best of our knowledge, the present study is the first to explore the etiology of EVD-like illnesses in uninfected patients in Sierra Leone, highlighting the importance of accurate diagnosis to EVD confirmation.

## INTRODUCTION

Since the first report in Sudan and Zaire in 1976, Ebola virus disease (EVD), a lethal illness with an average case fatality rate of 78% [1, 2], caused more than 30 outbreaks in the subsequent 40 years [3]. A new EVD outbreak was initiated in West Africa in February 2014 by a single spillover event followed by human-to-human transmission [1]. By November 2015, the 2014 EVD outbreak resulted in more than 28,600 infections and 11,300 deaths worldwide (http://www.who.int/csr/disease/ebola/en/). The global EVD epidemic sparked the participation of many countries in EVD surveillance of suspected subjects and expert training in clinical management [3]. In response to the requests of WHO and governments of the affected countries, Chinese government have deployed Chinese Military Medical Teams (CMMTs) to the areas and a total of 773 suspected and 285 confirmed EVD cases were diagnosed in Sierra Leone between 1 October 2014 and 22 March 2015 [4].

EVD is caused by Ebola virus (EBOV), one of five ebolaviruses in the Filoviridae family. The genome of EBOV consists of an enveloped, single-stranded, negative-sense RNA of about 19 kilobases [5]. Clinically, patients with EBOV infection present with signs or symptoms of fever or history of fever, intense fatigue or weakness, vomiting or nausea, and diarrhea [3, 6, 7]. Previous studies found significant differences in the clinical characteristics between EVD cases and non-cases [3, 6]. Centers for Disease Control and Prevention (CDC) defined EVD-suspected patients as exhibiting “elevated body temperature or subjective fever or symptoms, including severe headache, fatigue, muscle pain, vomiting, diarrhea, abdominal pain, or unexplained hemorrhage; and had an epidemiologic risk factor within the 21 days before the onset of symptoms” [8]. Suspected patients were confirmed based on the laboratory results of highly sensitive and specific methods, such as real-time reverse transcriptase (RT)-PCR [9, 10]. Based on current studies, only about half of all suspected EVD patients with clinical features or epidemiologic history were confirmed by laboratory test [6]. However, to the best of our knowledge, little is known regarding the etiology in EBOV-negative patients with EVD-like illnesses. Therefore, in this study, we explored the etiologies in EVD-like illnesses and compared the clinical features between EVD and non-EVD cases.

## RESULTS

### Study patients

During the study period, 883 suspected patients with significant symptoms and/or epidemiologic history were admitted to Jui Government Hospital, of whom 295 were confirmed with EBOV infections. Due to the limiting conditions in Sierra Leone, only 278 EBOV-negative patients were comprehensively screened for other illnesses, including 162 males (58.27%) and 116 females (41.73%). The median age of enrolled patients was 42.3 ± 8.6 years (range 2–75 years). Of the 278 patients, 202 reported an epidemiological history of illness (Table 1).

**Table 1:** Demographic and epidemical characteristics of Ebola virus (EBOV)-negative patients

Characteristics	EBOV-negative patients (*n* = 278)
**Age**	
Mean (y)	42.3 ± 8.6
Range (y)	2–75
**Gender**	
Male (%)	162 (58.27%)
Female (%)	116 (41.73%)
**Employment**	
Unemployed	103 (37.05%)
Trader	95 (34.17%)
Fisherman	56 (20.14%)
Children	11 (3.96%)
Transportation driver	5 (1.80%)
Teacher	2 (0.72%)
Unknown	6 (2.16%)
**Frequency of non-EVD illness**	
Malaria	121 (43.52%
HIV	44 (15.83%)
Lassa fever	36 (12.95%)
Tuberculosis	33 (11.87%)
Yellow fever	23 (8.27%)
Pneumonia	10 (3.60%)
Unknown	11 (3.96%)
**Epidemiologic history (within past 21 days)**	
Contact with EVD corpse	12 (4.32%)
Healthcare for EVD patients	31 (11.15%)
Contact with EVD-suspected patients	26 (9.35%)
Contact with clothes of EVD patients	56 (20.14%)
Contact with vomit/excrement of EVD patients	24 (8.63%)
Indirect contact with EVD patients	53 (19.07%)
Unknown	76 (27.34%)

### Clinical manifestations

The gap between symptom onset and hospital admission of EBOV-negative patients was 4 days (range 3–8 days). Upon admission, major symptoms reported included fever (223/278, 80.2%), headache (142/278, 51.1%), muscle and joint pain (123/278, 44.3%), fatigue (114/278, 41.1%), and nausea/vomiting (59/278, 21.2%) (Table 2).

**Table 2:** Comparison of the most common symptoms between EBOV-infected and non-infected patients presenting with EVD or EVD-like illness

Disease	Reference	Signs and symptoms in order of frequency						
		1st	2nd	3rd	4th	5th	6th	7th
EBOV-infected cases	Schieffelin et al. [7]	Fever (89%)	Headache (80%)	Weakness (66%)	Dizziness (60%)	Diarrhea (51%)	Abdominal pain (40%)	Vomiting (34%)
	Lado et al.[6]	Fever (84%)	Intense fatigue (73%)	Vomiting (55%)	Diarrhea (44%)	Anorexia (38%)	Abdominal pain (32%)	Conjunctivitis (30%)
	Bah et al. [17]	Fever (84%)	Fatigue (65%)	Diarrhea (62%)	Headache (57%)	Vomiting (57%)	Anorexia (43%)	
	Gao et al. [19]	Fever (83%)	Weakness (82%)	Loss of appetite (80%)	Cough (63%)	Headache (59%)	Joint pain (56%)	Abdominal pain (51%)
	Qin et al. [18]	Weakness (85%)	Fever (82%)	Headache (75%)	Muscle/joint pain (74%)	Extreme fatigue (56%)	Diarrhea (46%)	Vomiting (39%)
	Yan et al.[3]	Fatigue (85.2%)	Anorexia (84.3%)	Fever (75.9%)	Headache (72.2%)	Arthralgia (67.6%)	Nausea/vomiting (66.7%)	Myalgia (65.8%)
EBOV- non-infected cases in this study	Malaria	Fever (100%)	Headache (73.6%)	Joint pain (64.5%)	Fatigue (38%)	Nausea/vomiting (19%)	Diarrhea (5%)	Hemorrhage (0%)
	HIV	Fatigue (54.6%)	Fever (52.3%)	Joint pain (38.6%)	Diarrhea (34.1%)	Nausea/vomiting (25%)	Headache (11.4%)	Hemorrhage (4.6%)
	Lassa fever	Fever (72.2%)	Headache (61.1%)	Fatigue (44.5%)	Nausea/vomiting (33.3%)	Diarrhea (22.2%)	Hemorrhage (22.2%)	Joint pain (13.9%)
	Tuberculosis	Fever (63.6%)	Fatigue (33.3%)	Headache (18.2%)	Nausea/vomiting (15.2%)	Joint pain (12.1%)	Diarrhea (9.1%)	Hemorrhage (6.1%)
	Yellow fever	Fever (82.6%)	Headache (78.3%)	Joint pain (65.2%)	Fatigue (56.5%)	Nausea/vomiting (21.7%)	Diarrhea (13%)	Hemorrhage (0%)
	Pneumonia	Fever (80%)	Fatigue (20%)	Headache (10%)	Joint pain (10%)	Nausea/vomiting (10%)	Diarrhea (5%)	Hemorrhage (0%)

### Etiologies of EVD-like symptoms

Examination of EBOV-uninfected individuals for non-EVD illnesses was performed using different methods. Among the 278 EBOV-negative subjects, 121 (43.5%), 44 (15.6%), 36 (13.1%), 33 (11.7%), 23 (8.4%), and 10 (3.7%) were diagnosed with malaria, HIV, Lassa fever, tuberculosis, yellow fever, and pneumonia, respectively. Eleven patients remained undiagnosed (Table 1), which implied the possibility that fever of the patients were caused by non-infectious diseases. Table 2 lists the most common symptoms reported in EBOV-negative patients. Although no statistical analysis was performed, the clinical features of patients with non-EBV illness compared with EBOV infection seems to have no significant difference.

All patients were hospitalized for 1~11 days' worth of treatment according to respective diseases, and 13 (4.7%) patients died during the study. Six patients with malaria infection died due to septic shock (3), brain edema (2), or respiratory failure (1). Four patients with HIV infection died due to respiratory failure. One patient with Lassa fever died because of renal failure, and two undiagnosed patients died as a result of shock.

## DISCUSSION

The high lethality and contagiousness associated with the EVD outbreak of 2014 induced significant panic. The majority of cases of EVD occurred in Guinea, Sierra Leone, and Liberia [11], but most countries in the world established a series of measures to prevent and control the spread of EBOV infection. One of most important methods was to isolate suspected individuals and rapidly test for EBOV infection before discharge decision. The transmission of EBOV through contact posed a challenge to the prevention of cross infection between EBOV-infected and non-infected individuals during the outbreak. In addition, rapid diagnosis is also desirable to accommodate hospital patients with truly critical medical needs [11]. Thus, we examined the signs and symptoms of non-infected patients with EVD-like symptoms to determine whether EVD could be more rapidly distinguished based on clinical features.

A recent study reported that malaria, HIV, and tuberculosis are the most common neglected tropical diseases in Sub-Saharan Africa [12], and are also considered the “big three” infectious diseases worldwide [13]. These diseases, as well as other common infectious conditions, present similar clinical features to EVD. For example, Muhlberger and colleagues reported that common complaints of malaria patients involved fever, headache, fatigue, and musculo-skeletal symptoms [14]. Therefore, diseases manifested by symptoms of fever, fatigue, and headache would most likely mask cases of EVD, especially during mass outbreaks.

In the present study, 278 suspected patients, who were referred to holding centers based on the presence of EVD-like symptoms and/or epidemiologic history, were diagnosed as EBOV-negative, but tested positive for other infections. Malaria infections were the most frequently diagnosed, followed by HIV and Lassa fever. The top four symptoms reported for malaria, HIV, and Lassa fever included fever, headache, joint pain, and fatigue (Table 1). The integration of multiple previous studies suggests that the most common symptoms of EVD include fever, headache, fatigue or weakness, vomiting or nausea, diarrhea, and anorexia [3, 6, 7, 15–17]. Therefore, as concluded by Lado and colleagues [6], distinguishing EVD cases from suspected cases based on clinical symptoms is not sufficiently sensitive. However, our data imply that it would be of great value to rapidly test patients for malaria, HIV, and Lassa fever in order to discharge EBOV-negative patients. It should be noted that diseases diagnosed in EBOV-non-infected individuals, but who exhibit EVD-like symptoms, may vary according to local public health standards.

Another important point to be emphasized is the co-infection of five (1.8%) patients with both EBOV and malaria. These individuals were initially screened as EBOV-negative based on the RT-PCR test and were subsequently diagnosed with malaria, but in fact tested positive for EBOV on the third day of hospitalization. A possible reason for the false-negative cases could be the onset of an acute EBOV infection during patient referral or transportation. These results suggest that the presence of EBOV RNA should be re-tested in suspected patients with symptoms before discharge. Nonetheless, the rarity of co-infection events indicates that earlier confirmation of other diseases would greatly help stratify EBOV infections.

### Limitations

Several limitations need to be acknowledged. First, we screened for diseases in EBOV-negative patients by routine clinical methods. For example, the diagnosis of HIV infection was tested by ELISA, not Western blot, which is a confirmative/supplemental testing in antibody-based HIV testing algorithm in most countries [18, 19]. The screening techniques used in this study may therefore yield false-positive results. Second, we performed RT-PCR assays on blood samples, but not on other bodily fluids [20], which may produce potential false-negative results [21], albeit at a low rate. Third, occupations and epidemiologic history were provided by patients, which are subject to potential underreporting. However, underreporting to a certain degree was unavoidable in this clinical setting.

## MATERIALS AND METHODS

### EVD-suspected patients

From November 13, 2014 to March 16, 2015, patients with suspected EVD were admitted to Jui Government Hospital (Sierra Leone-China Friendship Hospital) in Freetown, Sierra Leone. Suspected cases of EVD were defined according to World Health Organization (WHO) standards [22]. Upon admission, patients filled out a case investigation form regarding personal information, diseases history, symptoms, and epidemiological history. In addition, written informed consent was obtained from all the participants of this study. Plasma from all EVD-suspected patients was tested for EBOV by *AccuPower*^®^ EBOV Real-Time RT-PCR Kit (Bioneer Corporation, Daejeon, Republic of Korea). All blood samples were treated and tested according to “Ebola hemorrhagic fever laboratory testing program (Chinese CDC)” [3, 23]. Patients testing negative for EBOV by RT-PCR were enrolled in the study.

### Diagnosis of EVD-like illnesses

EBOV-negative patients were spatially separated to avoid cross infection and further tested for malaria, yellow fever, Lassa fever, and HIV. Concomitant diseases that may influence the diagnosis were taken into account. Blood samples were screened for these illnesses independently by the Chinese CDC mobile laboratory team. Specifically, malaria was assayed with a rapid detection kit based on the colloidal gold method. Yellow fever was detected by Human yellow fever virus (YFV) antibody (IgM) ELISA Kit (MyBioSource, Inc., San Diego, USA) for specificity to IgM antibodies against yellow fever. Lassa fever was tested by ELISA for both Lassa fever antigen and virus-specific IgM. HIV was tested by the fourth-generation ELISA kit for both p24 antigen and HIV-1/2 antibody (Bio-Rad, Marnes-la-Coquette, France). To determine tuberculosis incidence and diagnosis of pneumonia, a chest X-ray was performed for the suspected tuberculosis patients presenting with low-grade fever and cough. The diagnosis of tuberculosis and pneumonia was verified by clinical symptoms, medical history, and chest X-ray. To avoid the possible window period of EBOV infection, RT-PCR assays for measuring plasma EBOV were re-conducted 72 hours after the first RT-PCR experiment. When appropriate, data were expressed as mean ± standard deviation (SD).

## CONCLUSIONS

In conclusion, we investigated the etiology of EVD-like illnesses in suspected patients who tested negative for EBOV in Sierra Leone during the 2014 EVD outbreak. Clinical features were not found to be significantly beneficial in distinguishing EVD from non-EVD illnesses. However, our study argues for more extensive research to determine the clinical and molecular characteristics uniquely representative of diseases associated with EBOV infection.
